# Geriatric nutrition risk index in the prediction of all-cause and cardiovascular mortality in elderly hypertensive population: NHANES 1999–2016

**DOI:** 10.3389/fcvm.2023.1203130

**Published:** 2023-07-03

**Authors:** Xuan Huo, Meiyin Wu, Dongmei Gao, YueShengzi Zhou, Xu Han, Weilin Lai, Mengqi Wang, Yilun Hang

**Affiliations:** ^1^ Department of Cardiology, Zhejiang Medical and Health Group Hangzhou Hospital, Zhejiang, China; ^2^ Department of Endocrinology, The First People's Hospital of Yuhang District, Hangzhou, China; ^3^ Department of Medical Oncology, Zhejiang Medical and Health Group Hangzhou Hospital, Zhejiang, China

**Keywords:** Geriatric nutritional risk index, hypertension, NHANES, observational study, mortality

## Abstract

**Background:**

Hypertension is a major risk factor for the global burden of disease, and nutrition is associated with an increased risk of mortality from multiple diseases. Few studies have explored the association of nutritional risk with all-cause mortality and cardiovascular mortality in hypertension, and our study aims to fill this knowledge gap.

**Method:**

We included data from the National Health and Nutrition Examination Survey (NHANES) from 1999 to 2016 on a total of 10,037 elderly patients with hypertension. The nutritional status was evaluated using the Geriatric Nutrition Risk Index (GNRI). Kaplan-Meier survival analysis was performed to analyze the survival rates of different nutritional risk groups. COX proportional risk regression models were used to analyze the predictive effect of GNRI on all-cause mortality and cardiovascular mortality in hypertensive patients. Restricted cubic splines (RCS) were used to explore the nonlinear relationship between GNRI and mortality.

**Result:**

The mean age of the hypertensive patients was 70.7 years. A total of 4255 (42.3%) all-cause mortality and 1207 (17.2%) cardiovascular mortality occurred during a median follow-up period of 106 months. Kaplan-Meier showed a more significant reduction in survival for the moderate to severe malnutrition risk of GNRI. The adjusted COX proportional hazards model showed that the hazard ratios for all-cause mortality and cardiovascular mortality in the moderate to severe malnutrition risk group for GNRI were 2.112 (95% CI, 1.377,3.240) and 2.604 (95% CI, 1.603,4.229), respectively. The RCS showed that increased GNRI was associated with a reduced risk of all-cause mortality and cardiovascular mortality risk reduction.

**Conclusion:**

Malnutrition exposure assessed by GNRI effectively predicts the risk of all-cause mortality and cardiovascular mortality in the elderly with hypertension.

## Introduction

Hypertension is a common chronic disease (1), with the World Health Organization (WHO) reporting that hypertension affects up to 40% of adults worldwide and that its prevalence continues to increase (2), thus hypertension has become a significant contributing risk factor to the global burden of disease (3). Prolonged elevated blood pressure leads to vascular endothelial dysfunction, sustained activation of inflammatory factors, and end-organ damage (4), therefore hypertension is significantly associated with cardiovascular disease (CVD), which causes approximately 7.5 million mortality worldwide (5).

Malnutrition is associated with the increasing prevalence of hypertension (6), and nutritional status is related to vascular endothelial damage repair, antioxidants, and antithrombosis (7). A systematic review showed that malnutrition was independently associated with increased length of ICU stay, ICU readmission rates, infection rates, and in-hospital mortality (8). To date, several studies have demonstrated that malnutrition is strongly associated with all-cause and cardiovascular mortality in patients (9–11). Some studies have shown an association between low body mass index and all-cause mortality in patients with hypertension (12). Hypertension and malnutrition can seriously affect the subjective quality of life, and life expectancy of hospitalized patients. The study (13) has shown that malnutrition shows a significant correlation with increased levels of the inflammatory response, and atherosclerosis progression, which may further increase the likelihood that it plays an important role in increasing cardiovascular-related events.

The Geriatric Nutrition Risk Index (GNRI), a simple nutritional assessment tool, is a new indicator to estimate geriatric internal medicine patients at high risk (14). Recent studies have shown that a high risk of malnutrition assessed by GNRI predicts the risk of death in patients with chronic kidney disease, cancer and diabetes (15–17). However, studies on GNRI as a predictor of all-cause mortality and cardiovascular mortality risk in hypertension are still limited, especially in the elderly. The United Nations defines people over 60 years of age as elderly (18). No study has yet validated the association between GNRI and all-cause and cardiovascular mortality in hypertensive patients, and our study aims to fill this knowledge gap.

## Methods

### Study design and study population

This retrospective cohort study was based on the National Health and Nutrition Examination Survey (NHANES) - a large national survey of the United States civilian population conducted by the Centers for Disease Control and Prevention's National Center for Health Statistics. The NHANES data were collected through a complex multi-stage probabilistic design that identifies strata based on geography and a certain percentage of the minority population (19). Furthermore, this study was supported by the National Center for Health Statistics Research Ethics Review Board, you can find it on this website: https://www.cdc.gov/nchs/nhanes/irba98.html. And the ethics approval numbers Protocol #98-12, Protocol #2005-06, Continuation of Protocol#2005-06, Protocol #2011-17and Continuation of Protocol#2011-2017. All participants provided written informed consent.

**Figure 1:**
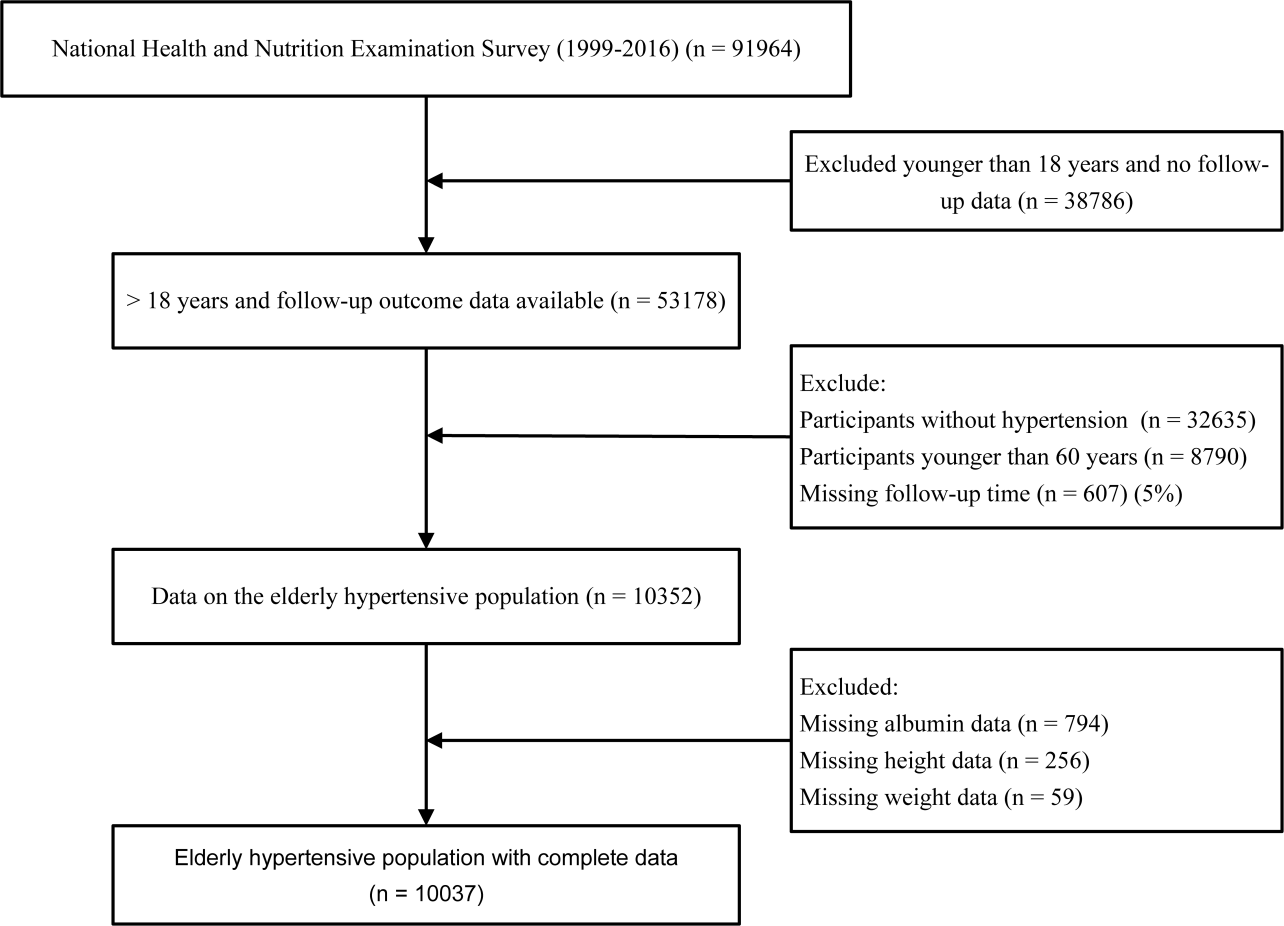
Flow diagram for the selection of the study population.

Data for the analysis were taken from NHANES 1999–2016 with a total of 91,964 participants. In the present study, we included people aged ≥60 years and excluded subjects without serum albumin, height, weight, follow-up data, and without hypertension disease. After applying the above criteria, 10,037 participants were finally included (Figure 1).

### Nutritional status assessment

The Geriatric Nutrition Risk Index was calculated as GNRI = [1.489 × serum albumin (g/L)] + (41.7 × weight (kg)/ideal weight (kg)) The ideal weight was calculated by the following Lorenz equation: 22 × square of height. If the patient's weight exceeded the ideal weight, the ratio of weight to ideal weight was set to 1 (14). Classification of patients was based on the following thresholds: moderate to severe risk of malnutrition: <92; low risk: 92 to 98; no risk: >98 (17).

### Definition of hypertension

A history of hypertension and antihypertensive medication was collected by questionnaire. Blood pressure was measured by a trained physician using a mercury sphygmomanometer with an appropriately sized cuff. Blood pressure measurements were performed three times and the average of the three measurements was defined as systolic blood pressure (SBP) and diastolic blood pressure (DBP). Hypertension was defined as having a self-reported history of hypertension or use of antihypertensive medication or SBP ≥ 140 mmHg or DPB ≥ 90 mmHg.

### Covariate assessments

We included patient demographic information, including age, gender, race, marital status, and education levels. Other covariates selected from previous literature reports or clinical experience that had an impact on the hypertensive population included body mass index (BMI), lymphocytes, neutrophils, serum creatinine, serum uric acid, triglycerides, fasting glucose, high density lipoprotein cholesterol (HDL-C), low density lipoprotein cholesterol (LDL-C), serum albumin, C-reactive protein (CRP), sodium and salt intake, smoking, alcohol consumption, physical activity, diabetes, cardiovascular disease, and chronic kidney disease (20). Race was categorized as Mexican American, non-Hispanic white, non-Hispanic black, or other. Education levels were classified as less than 9th Grade, and higher than 9th Grate or 9th Grate. Marital status was categorized as with partner (married or living with a partner), without partner (widowed, divorced, or separated), or never married. Body mass index (BMI) was calculated by the following formula: BMI = weight (kg)/height^2^ (m^2^) (21). The sodium and salt intake were assessed from a 24-hour recall questionnaire (22). According to the median sodium and salt intake, there were “high sodium and salt intake” and “low sodium and salt intake” groups. Participants were categorized as “mild”, “moderate or heavy”, “former” and “never” based on the number of drinks per day he/she had drunk (23). Participants who were “mild” were considered to be drinking alcohol ≤1 drink in women and ≤2 drinks in men; Participants who were “heavy” were considered to be drinking alcohol ≤2 drinks in women and ≤3drinks in men or individuals had drunk ≥3 drinks of woman and ≥4drinks of man; Participants who used to drink alcohol but do not drink now are defined as “former” and never drink alcohol is defined as “never”. Smoking status was defined as the number and timeline of cigarettes in life (never, smoked less than 100 cigarettes; former, smoked more than 100 cigarettes in life and smoke not at all now; now, smoked more than 100 cigarettes in life and smoke some days or every day). Physical activity was classified as ideal [≥8,000 metabolic equivalent task (MET) minutes/week], intermediate (600–7,999 MET minutes/week), and poor (<600 MET minutes/week) (24). Definition of cardiovascular disease: data were obtained from self-report data from the personal interview: “Have you ever been told by a doctor or other health professional that you have CHF (Congestive Heart Failure)/CHD (Coronary Heart Disease) /angina/heart attack/stroke?” If any of the above questions were answered in the affirmative, CVD was considered to be present. Glomerular filtration rate (eGFR) was estimated using the Chronic Kidney Disease Epidemiology Collaboration (CKD-EPI-2009) creatinine equation (25). Chronic kidney disease (CKD) was defined as estimated eGFR < 60 ml/min/1.73 m^2^ (26). Diabetes mellitus was defined as meeting one of the following conditions: the doctor told you have diabetes, glycohemoglobin (HbA1c) (%) > 6.5, fasting glucose (mmol/L) ≥ 7.0, random blood glucose (mmol/L) ≥ 11.1, 2-hour oral glucose tolerance test (OGTT) blood glucose (mmol/L) ≥ 11.1 and use of diabetes medication or insulin. Furthermore, we categorized the use of antihypertensive medications according to the latest American College of Cardiology/American Heart Association (ACC/AHA) guidelines for hypertension (27). These included Beta blockers, Calcium channel blockers, Angiotensin-Converting Enzyme Inhibitors/Angiotensin II receptor antagonists (ACEI/ARB), and Diuretics.

### Statistical analysis

Statistical analyses of data from this study were performed using R software (The R Foundation; http://www.r-project.org; version 4.2.2). NHANES created weights to account for the complex survey design, survey nonresponse, and post-stratification adjustments to match the total population residing in the United States. Therefore, in all the following analyses, we weighted the samples according to NHANES analysis guidelines (28).

Initially, the baseline characteristics of patients were compared by dividing the sample into three groups: no-risk, low-risk, and moderate-to-severe risk of adverse events according to GNRI thresholds. Continuous variables were expressed as weighted means ± standard deviations. Differences between the 3 study groups were analyzed by One-way analysis of variance (ANOVA). Categorical variables were expressed as numbers and percentages and compared using the Rao-Scott chi-squared test. NHANES public-use linked mortality file data as of December 31, 2019 were used to determine the mortality status of the follow-up population (29).Survival rates for different nutritional risk groups were then analyzed using the Kaplan-Meier survival analysis. Furthermore, Cox proportional hazard regression models were used to estimate hazard ratios (HRs) and 95% confidence intervals (CIs) for cardiovascular mortality and all-cause mortality. The nonlinear relationship between GNRI and the presence of all-cause mortality and cardiovascular mortality was examined using a multivariate-adjusted Cox restricted cubic spline regression model. Finally, subgroup analyses were performed stratified by sodium and salt intake, physical activity, cardiovascular disease, diabetes mellitus, and chronic kidney disease. In all of these analyses, a two-sided *P* < 0.05 was considered statistically significant.

## Results

### Baseline characteristics

A total of 10,037 elderly patients with hypertension were included in this study. Based on the calculation of the weights for each sample, our sample represents approximately 3,322,957 elderly hypertensive patients in the United States. The median follow-up time was 106 months. Baseline characteristics of the total population and groups according to different GNRI levels are listed in the table (Table 1). In the total population, the mean age of patients was 70.7 years and the mean GNRI was 104.1. Among the 3 groups compared by GNRI classification, age, lymphocyte count, neutrophil count, serum creatinine, triglycerides, LDL cholesterol, C-reactive protein, diastolic blood pressure, gender, race, marital status, cardiovascular disease, diabetes mellitus, chronic kidney disease, smoking, and alcohol consumption were statistically different (*P* < 0.05). Participants in the moderate to severe malnutrition risk group of GNRI (GNRI < 92) had higher lymphocytes, C-reactive protein levels, higher prevalence of diabetes, and chronic kidney disease. In addition, BMI, serum uric acid, HDL cholesterol, and systolic blood pressure were not statistically different (*P* > 0.05).

**Table 1:** Baseline characteristics of the elderly hypertensive population by GNRI groups.

Characteristics	Overall	M/S risk	Low risk	No risk	*P*-value
	*N* = 10,037	*N* = 162	*N* = 825	*N* = 9,050	
GNRI	104.1 ± 0.1	88.2 ± 0.4	95.6 ± 0.1	104.9 ± 0.1	<0.001
Age, year	70.7 ± 0.1	71.2 ± 0.8	73.0 ± 0.4	70.5 ± 0.1	<0.001
BMI, kg/m^2^	29.5 ± 0.1	27.9 ± 1.1	29.3 ± 0.4	29.5 ± 0.1	0.335
Lymphocyte, 10^9^/L	2.0 ± 0.0	1.8 ± 0.1	1.9 ± 0.0	2.0 ± 0.0	0.005
Neutrophil, 10^9^/L	4.3 ± 0.0	5.2 ± 0.2	4.8 ± 0.1	4.3 ± 0.0	<0.001
Cr, μmol/L	88.8 ± 0.6	136.4 ± 12.9	98.1 ± 2.2	87.4 ± 0.6	<0.001
UA, μmol/L	345.3 ± 1.3	333.1 ± 10.1	347.0 ± 3.8	345.4 ± 1.3	0.377
TG, mmol/L	1.7 ± 0.0	1.5 ± 0.1	1.6 ± 0.1	1.7 ± 0.0	0.043
TC, mmol/L	5.1 ± 0.0	4.7 ± 0.1	5.1 ± 0.0	4.8 ± 0.0	<0.001
HDL-C, mmol/L	1.4 ± 0.0	1.4 ± 0.0	1.4 ± 0.0	1.4 ± 0.0	0.384
LDL-C, mmol/L	2.9 ± 0.0	2.6 ± 0.1	2.7 ± 0.0	2.9 ± 0.0	<0.001
Albumin, g/L	42.1 ± 0.1	32.9 ± 0.3	37.0 ± 0.1	42.6 ± 0.0	<0.001
CRP, mg/dl	0.4 ± 0.0	1.7 ± 0.2	0.9 ± 0.1	0.4 ± 0.0	<0.001
SBP, mmHg	139.1 ± 0.3	141.6 ± 2.0	140.4 ± 1.0	139.0 ± 0.4	0.233
DBP, mmHg	68.5 ± 0.2	66.1 ± 1.3	65.5 ± 0.7	68.7 ± 0.2	<0.001
Sodium and salt intake, mg	2,965.7 ± 20.2	2,782.8 ± 114.1	2,768.3 ± 61.0	2,983.5 ± 21.2	0.002
eGFR, ml/min/1.73 m^2^	70.7 ± 0.3	59.4 ± 2.8	71.2 ± 0.3	65.5 ± 1.0	<0.001
Gender					<0.001
Male	4,727 (47.1)	74 (41.8)	317 (30.3)	4,336 (43.6)	
Female	5,310 (52.9)	88 (58.2)	508 (69.7)	4,714 (56.4)	
Race					<0.001
Non-Hispanic white	5,181 (51.6)	60 (66.1)	377 (70.0)	4,744 (79.1)	
Non-Hispanic black	2,110 (21)	52 (17.9)	258 (18.0)	1,800 (8.9)	
Mexican American	1,515 (15.1)	26 (5.0)	100 (4.1)	1,389 (3.9)	
Other race	1,231 (12.3)	24 (11.0)	90 (7.9)	1,117 (8.0)	
Marital status					<0.001
With-partner	5,653 (56.3)	79 (51.7)	357 (46.9)	5,217 (62.3)	
Without-partner	3,841 (38.3)	73 (45.6)	423 (48.9)	3,345 (33.3)	
Never married	543 (5.4)	10 (2.6)	45 (4.3)	488 (4.4)	
Education					0.067
High school or above	7,977 (79.5)	122 (83.7)	655 (86.8)	7,200 (88.9)	
Less than High School	2,060 (20.5)	40 (16.3)	170 (13.2)	1,850 (11.1)	
Cardiovascular Disease					<0.001
Yes	2,827 (28.2)	61 (36.0)	310 (38.7)	2,456 (26.4)	
No	7,210 (71.8)	101 (64.0)	515 (61.3)	6,594 (73.6)	
Diabetes					<0.001
Yes	3,516 (35)	79 (40.7)	335 (36.5)	3,102 (29.9)	
No	6,521 (65)	83 (59.3)	490 (63.5)	5,948 (70.1)	
Chronic kidney disease					<0.001
Yes	4,264 (42.5)	105 (62.3)	462 (54.8)	3,697 (38.5)	
No	5,773 (57.5)	57 (37.7)	363 (45.2)	5,353 (61.5)	
Physical Activity					0.006
Poor	4,263 (42.5)	72 (44.0)	394 (48.7)	3,797 (39.7)	
Intermediate	5,250 (52.3)	80 (49.8)	390 (46.2)	4,780 (54.8)	
Ideal	524 (5.2)	10 (6.3)	41 (5.1)	473 (5.5)	
Drinking					<0.001
Never	2,359 (23.5)	51 (26.1)	233 (27.3)	2,075 (20.5)	
Former	2,910 (29)	54 (29.1)	282 (34.3)	2,574 (25.2)	
Mild	3,267 (32.5)	37 (29.0)	205 (26.3)	3,025 (38.8)	
Moderate or heavy	1,501 (15)	20 (15.8)	105 (12.1)	1,376 (15.6)	
Smoking					<0.001
Never	4,909 (48.9)	59 (30.7)	358 (42.5)	4,492 (49.0)	
Former	3,951 (39.4)	72 (49.1)	318 (40.8)	3,561 (40.8)	
Now	1,177 (11.7)	31 (20.2)	149 (16.7)	997 (10.2)	
Beta-blockers					<0.001
Yes	3,086 (30.7)	70 (46.3)	315 (40.9)	2,701 (31.3)	
No	6951 (69.3)	92 (53.7)	510 (59.1)	6,349 (68.7)	
Calcium channel blockers					0.626
Yes	2,691 (26.8)	46 (24.8)	231 (26.5)	2,414 (24.7)	
No	7,346 (73.2)	116 (75.2)	594 (73.5)	6,636 (75.3)	
ACEI/ARB					0.006
Yes	4,962 (49.4)	71 (38.3)	431 (54.4)	4,460 (50.7)	
No	5,075 (50.6)	91 (61.7)	394 (45.6)	4,590 (49.3)	
Diuretics					0.059
Yes	3,668 (36.5)	61 (41.5)	329 (41.9)	3,278 (36.9)	
No	6,369 (63.5)	101 (58.5)	496(58.1)	5,772(63.1)	
Statins					0.031
Yes	3,936(39.2)	49(32.6)	300(37.9)	3,587(42.2)	
No	6,101(60.8)	113(67.4)	525(62.1)	5,463(57.8)	

Enumeration data are presented as weighted means ± standard deviations and measurement data as percentages.

GNRI, geriatric nutrition risk index; BMI, body mass index; Cr, creatinine; UA, uric acid; TG, triglyceride; TC, Total cholesterol; LDL-C, low-density lipoprotein cholesterol; HDL-C, high-density lipoprotein cholesterol; CRP, C-reactive protein; eGFR, glomerular filtration rate; SBP, systolic blood pressure; DBP, diastolic blood pressure; ACEI/ARB, Angiotensin-Converting Enzyme Inhibitors/Angiotensin II receptor antagonists.

### The relationship between geriatric nutritional risk Index and mortality

**Figure 2:**
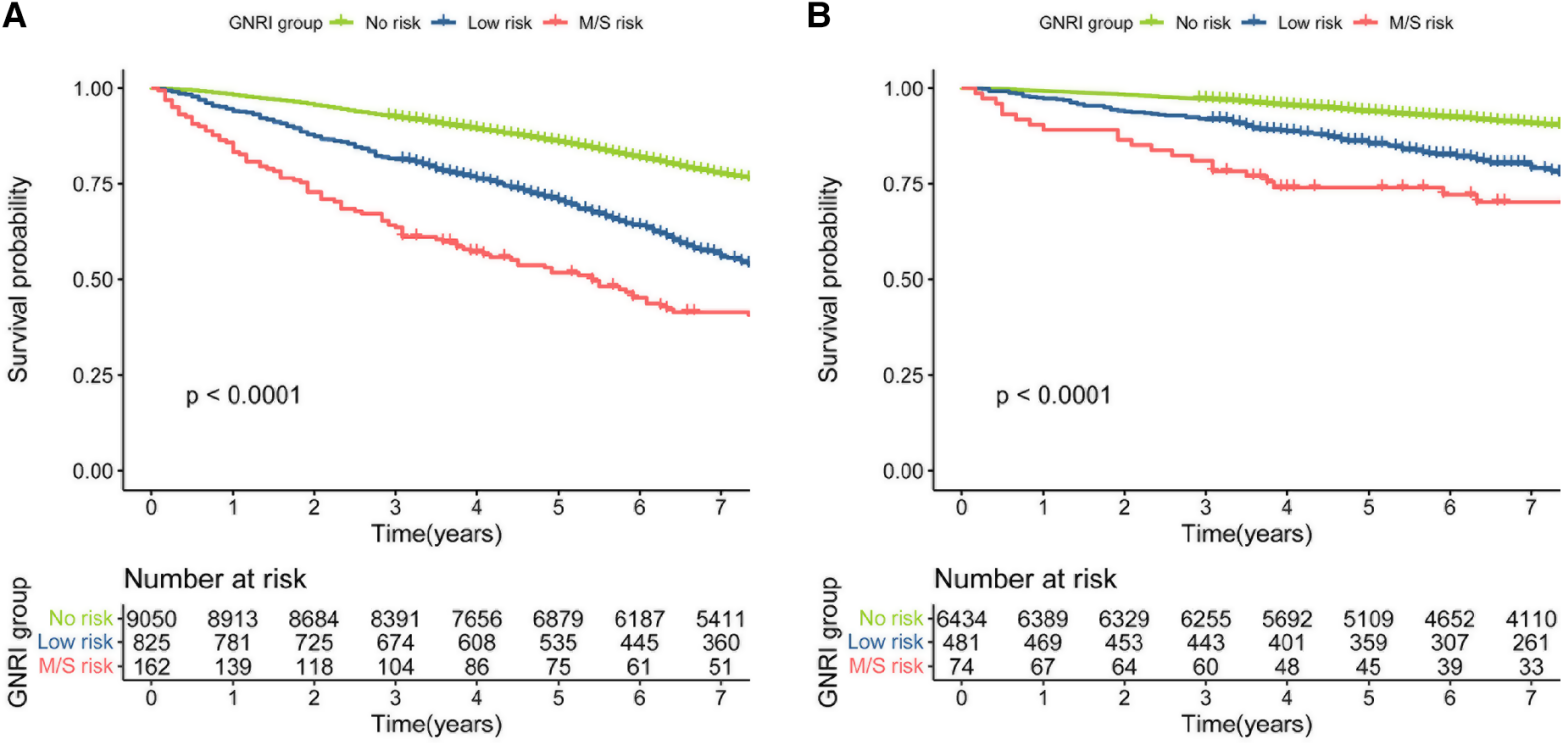
(**A**) kaplan–meier survival curve for all-cause mortality. (**B**) Kaplan–Meier survival curve for cardiovascular mortality.

During a median follow-up period of 106 months, 4255 (42.3%) all-cause mortality and 1207 (17.2%) cardiovascular disease mortality occurred in patients with hypertension. We used Kaplan-Meier to analyze survival between different groups at moderate to severe risk of malnutrition, low risk, and no risk of GNRI. The results showed that different levels of GNRI differed between all-cause and cardiovascular mortality (log-rank of trend *p* < 0.0001). The reduction in survival was more significant for moderate to severe malnutrition risk of GNRI for both all-cause and cardiovascular mortality (Figure 2).

In the COX proportional hazard regression model, GNRI is associated with the risk of all-cause mortality and cardiovascular mortality. In model 1, no variables were adjusted for. Elevated GNRI was associated with reduced risk of all-cause mortality and cardiovascular mortality [(HR, 0.941, 95% CI 0.933, 0.949) and [HR = 0.935, 95% CI (0.922, 0.949), respectively]. Similar findings were found for the adjusted Models 2, 3, and 4. And in the fully adjusted model 4, GNRI had hazard ratios of 0.958 (95% CI, 0.949, 0.967), and 0.956 (95% CI, 0.941, 0.972) for all-cause mortality and cardiovascular mortality, respectively. When GNRI was used as a categorical variable, the moderate to severe malnutrition risk of GNRI was shown to be associated with the risk of all-cause mortality and cardiovascular mortality in the unadjusted model with hazard ratios of 2.730 (95% CI, 2.006,3.714) and 2.891 (95% CI, 1.639,5.101), respectively. In the fully adjusted model (model 4), the hazard ratios for the risk of moderate to severe malnutrition in GNRI were 2.112 (95% CI, 1.377, 3.240) and 2.604 (95% CI, 1.603,4.229) for all-cause mortality and cardiovascular mortality, respectively (Table 2).

**Table 2:** COX proportional hazard regression analysis of GNRI and all-cause mortality and cardiovascular mortality in an elderly hypertensive population.

	Model 1		Model 2		Model 3		Model 4	
	HR 95% CI	*P*-value	HR 95% CI	*P*-value	HR 95% CI	*P*-value	HR 95% CI	*P*-value
All-cause mortality								
GNRI	0.941 (0.933,0.949)	<0.001	0.953 (0.944,0.962)	<0.001	0.958 (0.949,0.967)	<0.001	0.958 (0.949,0.967)	<0.001
GNRI (Category)								
No risk	Reference		Reference		Reference		Reference	
Low risk	2.138 (1.862,2.454)	<0.001	1.759 (1.537,2.013)	<0.001	1.613 (1.407,1.850)	<0.001	1.609 (1.403,1.845)	<0.001
M/S risk	2.730 (2.006,3.714)	<0.0001	2.442 (1.553,3.842)	<0.001	2.126 (1.370,3.300)	<0.001	2.112 (1.377,3.240)	<0.001
P for trend		<0.001		<0.001		<0.001		<0.001
Cardiovascular mortality								
GNRI	0.935 (0.922,0.949)	<0.001	0.948 (0.933,0.963)	<0.001	0.956 (0.941,0.972)	<0.001	0.956 (0.941,0.972)	<0.001
GNRI (Category)								
No risk	Reference		Reference		Reference		Reference	
Low risk	2.197 (1.756,2.749)	<0.001	1.634 (1.277,2.090)	<0.001	1.442 (1.124,1.850)	0.004	1.463 (1.143,1.873)	0.003
M/S risk	2.891 (1.639,5.101)	<0.001	3.120 (1.872,5.201)	<0.001	2.725 (1.685,4.407)	<0.001	2.604 (1.603,4.229)	<0.001
P for trend		<0.001		<0.001		<0.001		<0.001

Model 1 adjusted for none. Model 2 adjusted for age, gender, race, marital status, education, diabetes mellitus, cardiovascular disease, chronic kidney disease, diastolic blood pressure, systolic blood pressure, physical activity, smoking, and drinking. Model 3 adjusted for Model 2 + lymphocytes, neutrophils, eGFR, uric acid, triglycerides, HDL, LDL, and C-reactive protein. Model 4 adjusted for Model 3 + medication (beta-blockers, calcium channel blockers, ACEI/ARB, diuretics, and statins).

### Nonlinear association between GNRI and all-cause and cardiovascular mortality

**Figure 3:**
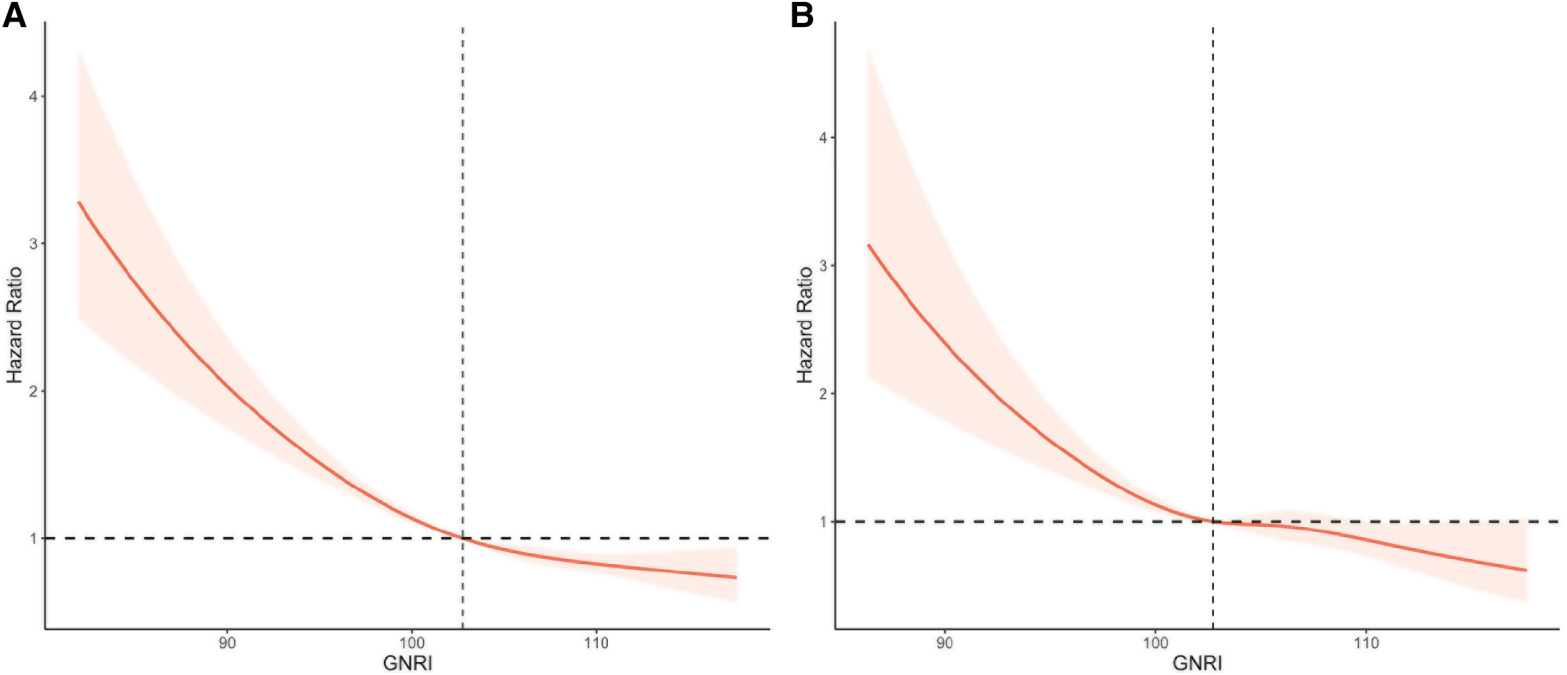
Restricted cubic splines of GNRI and mortality in elderly patients with hypertension. (**A**) All-cause mortality. (**B**) Cardiovascular mortality. Adjusted for age, gender, race, marital status, education, diabetes mellitus, cardiovascular disease, chronic kidney disease, diastolic blood pressure, systolic blood pressure, physical activity, smoking, drinking, lymphocytes, neutrophils, creatinine, uric acid, triglycerides, HDL, LDL, C-reactive protein, and medication (beta-blockers, calcium channel blockers, ACEI/ARB, diuretics, and statins).

In a restricted cubic spline regression model (RCS) fully adjusted for confounders, the increase in GNRI was associated with a reduced risk of all-cause mortality and cardiovascular mortality in the elderly hypertensive population, but they both showed a non-linear trend (non-linear *p* < 0.001 and non-linear *p* = 0.015, respectively) (Figure 3).

### Subgroup analysis

We examined the association between GNRI and all-cause mortality and cardiovascular mortality in the subgroup analysis (Table 3). The results showed that GNRI interacted with sodium and salt intake and physical activity in all-cause mortality. For cardiovascular mortality, there was no interaction between GNRI and sodium and salt intake, physical activity, cardiovascular disease, diabetes mellitus, and chronic kidney disease. In addition, GNRI was not associated with all-cause mortality and cardiovascular mortality in an older hypertensive population in which physical activity was ideal (*P* > 0.05).

**Table 3:** Subgroup analysis of the association with GNRI (classification) and all-cause mortality and cardiovascular mortality in a hypertensive population.

Subgroups	Number (%)	All-cause mortality		*p* for interaction	Cardiovascular mortality		*p* for interaction
		Low risk vs. No risk	M/S risk vs. No risk		Low risk vs. No risk	M/S risk vs. No risk	
Sodium				0.027			0.398
Low	5,019 (50.0)	1.973 (1.593,2.444)***	3.563 (2.455,5.171)***		2.061 (1.407,3.017)***	3.729 (1.941,7.165)***	
High	5,018 (50.0)	1.498 (1.223,1.834)***	2.043 (1.150,3.630)***		1.544 (1.098,2.170)*	2.117 (0.829,5.411)**	
MET				0.002			0.173
Poor	4,263 (42.5)	1.661 (1.402,1.969)***	1.645 (1.004,2.695)***		1.469 (1.058,2.041)*	2.413 (1.067,5.457)**	
Intermediate	5,250 (52.3)	1.884 (1.488,2.385)***	4.482 (3.239,6.202)***		2.388 (1.720,3.314)***	3.566 (1.839,6.912)***	
Ideal	524 (5.2)	1.520 (0.762, 3.031)	2.238 (0.478,10.482)		0.615 (0.176, 2.153)	1.325 (0.072,24.332)	
Cardiovascular Disease				0.064			0.369
Yes	2,827 (28.2)	1.777 (1.460,2.164)***	1.906 (1.038,3.502)***		1.997 (1.477,2.700)***	3.121 (1.512,6.444)***	
No	7,210 (71.8)	1.625 (1.330,1.985)***	3.136 (2.058,4.777)***		1.441 (1.009,2.058)*	2.656 (1.188,5.937)**	
Diabetes				0.215			0.784
Yes	3,516 (35)	1.831 (1.442,2.327)***	3.200 (1.989,5.147)***		1.595 (1.030,2.470)*	2.497 (0.910,6.847)**	
No	6,521 (65)	1.627 (1.361,1.945)***	2.302 (1.434,3.696)***		1.782 (1.296,2.450)***	2.890 (1.404,5.949)***	
Chronic kidney disease				0.501			0.222
Yes	4,264 (42.5)	1.692 (1.455,1.968)***	2.236 (1.466,3.409)***		1.845 (1.387,2.455)***	3.177 (1.775,5.687)***	
No	5,773 (57.5)	1.663 (1.286,2.151)***	2.946 (1.681,5.161)***		1.367 (0.802,2.329)	1.637 (0.563,4.758)	

Significance Marker:

* *p* < 0.05.

** *p* < 0.01.

*** *p* < 0.001.

Adjusted for age, gender, race, marital status, education, smoking, drinking, and medication (beta-blockers, calcium channel blockers, ACEI/ARB, diuretics, and statins).

## Discussion

In this large retrospective cohort study, we included 10,037 elderly hypertensive individuals and assessed their nutritional status using the GNRI. Our sample size represented a population of approximately 33,227,957 older adults with hypertension in the United States. We first analyzed the survival rates of all-cause mortality and cardiovascular mortality in hypertensive patients with different nutritional risks using Kaplan-Meier. In addition, COX proportional hazard regression models revealed that GNRI-assessed risk of poor nutrition was associated with increased risk of all-cause mortality and cardiovascular mortality in the hypertensive population. Further, restricted cubic spline regression models revealed a nonlinear trend of GNRI with all-cause mortality and cardiovascular mortality. Finally, subgroup analysis showed that GNRI was associated with risk of mortality in different subgroups of the elderly hypertensive population.

Nutritional status is a particularly important factor in predicting mortality from various diseases (30, 31). Long-standing chronic diseases, such as hypertension, diabetes, and coronary heart disease, can cause malnutrition, exacerbate disease progression and affect patient prognosis (32, 33). The effect of nutritional status on the prevalence of hypertension and its prognosis has been demonstrated in several studies. A cohort study from rural China confirmed that low body mass index was associated with all-cause mortality in hypertensive patients (12). However, some studies have also shown a U-shaped relationship between BMI and mortality in hypertension (34). For overweight or obese individuals does not mean that there is adequate nutrition in the body (35). On the contrary, fat accumulation and obesity can also indirectly lead to further nutritional disorders in patients through metabolic and organismal composition changes and through acute and chronic diseases that can adversely affect the nutritional status (36). Therefore, GNRI nicely combines BMI with serum albumin levels to assess a person's nutritional status. Sun et al. showed that nutritional status as assessed by Controlling Nutritional Status (CONUT) was associated with mortality in elderly patients with hypertension (37). A study of nutritional status as assessed by the Mini Nutritional Assessment Short-Form (MNA-SF) confirmed that high MNA-SF improves survival in hypertensive patients (38). However, BMI levels may vary significantly between regions and age groups (39). CONUT is assessed by serum cholesterol and may be influenced by medications. In contrast, the MNA-SF assessment may be more complex. Currently, GNRI assessed by serum albumin and BMI has been used extensively for prediction of diseases such as oncology, coronary heart disease, etc. It is simple to calculate and relatively easy to obtain. To our knowledge, no study has separately examined the relationship between GNRI and mortality among elderly patients with hypertension. Our study suggests that GNRI could be a fine predictor of prognosis in elderly patients with hypertension.

In this study, Kaplan-Meier curve analysis showed that both moderate to severe risk and low risk of poor nutrition with GNRI were associated with increased risk of all-cause mortality and cardiovascular mortality in hypertensive patients. To exclude other potential risk factors and comorbidities, we used Cox regression analysis to assess the independent relationship between GNRI and mortality in patients with hypertension. In the fully adjusted model, the hazard ratios for moderate to severe malnutrition risk of GNRI in all-cause mortality and cardiovascular mortality were 2.112(95% CI, 1.377,3.240) and 2.604(95% CI, 1.603,4.229), respectively. These results suggest that the risk of moderate to severe malnutrition in GNRI is an independent predictor of mortality in hypertensive patients. This is consistent with the results on nutritional status and risk of mortality in hypertensive patients in other populations, although they are using different nutritional scores (40–42). Numerous studies have shown that GNRI is reproducible and a more effective predictor of nutritional risk factors and prognosis than BMI and serum albumin alone (14, 43). In addition, the use of computerized GNRI calculation allows for screening of high nutritional risk groups and facilitates long-term, regular, large sample monitoring and follow-up (17).

The results of this observational study are difficult to reveal the specific mechanisms of how malnutrition affects all-cause mortality and cardiovascular mortality in hypertensive patients, but it may suggest the following mechanisms. Low nutritional status is associated with decreased immunity of the organism, which in the elderly population will lead to increased susceptibility to disease, which adds to the disease burden (44). It has been shown that inflammatory factor expression is significantly upregulated in hypertensive patients (45), such as IL-6 and TNF-α, and malnutrition is associated with the activation of inflammatory factors (7). These underlying inflammations may lead to vascular endothelial dysfunction as well as excessive proliferation and migration of vascular smooth muscle cells (46), which in turn aggravate the risk of developing some cardiovascular diseases such as atherosclerosis, coronary heart disease, and heart failure, ultimately leading to cardiovascular events (47). In addition, specific micronutrient deficiencies, such as B vitamins, vitamin D, and dietary fiber may coexist with malnutrition and promote the progression of hypertension (48–50).

The main strengths of this study are as follows: first, this study is a large sample size from a well-established national cohort of the US National Health and Nutrition Examination Survey. Second, we weighted all statistical analyses to obtain reliable results. However, there are still some limitations of this study; first, this study is a retrospective study and there may be unknown confounding factors. Secondly, GNRI was assessed only once in this study, and its change over time during the follow-up period was not assessed. Thirdly, we did not take into account the duration of hypertension.

## Conclusion

Calculation of GNRI by using common laboratory indicators provides a practical tool for the first screening of nutritional risk in hypertensive patients, and malnutrition exposure assessed by GNRI can effectively predict the risk of all-cause mortality and cardiovascular mortality in the elderly with hypertension.

## Data Availability

The raw data supporting the conclusions of this article will be made available by the authors, without undue reservation.
